# Evolution towards Virulence in a *Burkholderia* Two-Component System

**DOI:** 10.1128/mBio.01823-21

**Published:** 2021-08-10

**Authors:** Matthew M. Schaefers, Benjamin X. Wang, Nicole M. Boisvert, Sarah J. Martini, Sarah L. Bonney, Christopher W. Marshall, Michael T. Laub, Vaughn S. Cooper, Gregory P. Priebe

**Affiliations:** a Division of Critical Care Medicine, Department of Anesthesiology, Critical Care and Pain Medicine, Boston Children’s Hospital, Boston, Massachusetts, USA; b Harvard Medical School, Boston, Massachusetts, USA; c Department of Biology, Massachusetts Institute of Technology, Cambridge, Massachusetts, USA; d Microbiology and Molecular Genetics, University of Pittsburgh, Pittsburgh, Pennsylvania, USA; e Howard Hughes Medical Institute, Massachusetts Institute of Technology, Cambridge, Massachusetts, USA; Emory University School of Medicine

**Keywords:** *Burkholderia*, evolution, two-component regulatory systems, virulence regulation

## Abstract

Bacteria in the Burkholderia cepacia complex (BCC) are significant pathogens for people with cystic fibrosis (CF) and are often extensively antibiotic resistant. Here, we assess the impacts of clinically observed mutations in *fixL*, which encodes the sensor histidine kinase FixL. FixL along with FixJ compose a two-component system that regulates multiple phenotypes. Mutations in *fixL* across two species, B. dolosa and B. multivorans, have shown evidence of positive selection during chronic lung infection in CF. Herein, we find that BCC carrying the conserved, ancestral *fixL* sequence have lower survival in macrophages and in murine pneumonia models than mutants carrying evolved *fixL* sequences associated with clinical decline in CF patients. *In vitro* phosphotransfer experiments found that one evolved FixL protein, W439S, has a reduced ability to autophosphorylate and phosphorylate FixJ, while LacZ reporter experiments demonstrate that B. dolosa carrying evolved *fixL* alleles has reduced *fix* pathway activity. Interestingly, *B. dolosa* carrying evolved *fixL* alleles was less fit in a soil assay than those strains carrying the ancestral allele, demonstrating that increased survival of these variants in macrophages and the murine lung comes at a potential expense in their environmental reservoir. Thus, modulation of the two-component system encoded by *fixLJ* by point mutations is one mechanism that allows BCC to adapt to the host infection environment.

## INTRODUCTION

The Burkholderia cepacia complex (BCC) is a group of more than 20 species of closely related Gram-negative bacilli that can be dangerous respiratory pathogens for people with cystic fibrosis (CF) (1, 2). B. cenocepacia and B. multivorans are the most common species of BCC seen in infections among CF patients in the United States, although there is significant variability based on geographic region and institution (3–7), and *B. multivorans* has emerged as the predominant BCC species infecting CF patients in some regions (4–7). BCC members have caused several outbreaks within the CF community (2), including one outbreak of a highly antibiotic-resistant strain of *B. dolosa* among almost 40 CF patients in Boston (8) and another of B. cenocepacia in Toronto (9). BCC can also cause serious infections in individuals with chronic granulomatous disease (CGD) (10). Outbreaks of hospital-acquired BCC infections in non-CF and non-CGD patients have also been increasingly described and are often associated with contaminated medications (11–16), including a recent outbreak associated with contamination of the stool softener docusate with B. contaminans (15, 16).

Analysis of genomic diversity arising during *B. dolosa* and *B. multivorans* chronic infections in CF identified the two-component system (TCS) encoded by *fixLJ* as a pathway that is under positive selection, evident from genetic parallelism among many independent infections (17–19). TCSs are one mechanism that bacteria use to sense and respond to their environment (20). Our previous work determined that the TCS encoded by *fixLJ* senses oxygen depletion, is important for virulence in a murine model of pneumonia, and regulates ∼11% of the genome (21). Additionally, we found that the *fixLJ* system is involved in biofilm formation and motility as *B. dolosa* lacking *fixLJ* made more biofilm and had reduced motility (21). The TCS encoded by *fixLJ* was also critical for survival within THP-1-derived human macrophages. These experiments were conducted using a *fixLJ* deletion mutant to determine the effects of deletion of both genes. However, mutations observed in the clinic generate phenotypes that are more nuanced than these large deletions (22). Understanding how these clinically observed mutations alter pathogen phenotypes can inform treatment and prevention regimens. In the current study, we found that mutants carrying clinically observed *fixL* mutations (encoding single amino acid changes) were more virulent, had altered gene expression, and were less able to survive within soil than otherwise isogenic mutants carrying ancestral *fixL* variants. These results highlight the importance of the TCS pathway encoded by *fixLJ* in BCC pathogenesis and provide insight into the evolution of the BCC during chronic infection.

## RESULTS

### Mutations within the predicted sensory domain of BCC *fixL* are associated with a decline in lung function.

**FIG 1:**
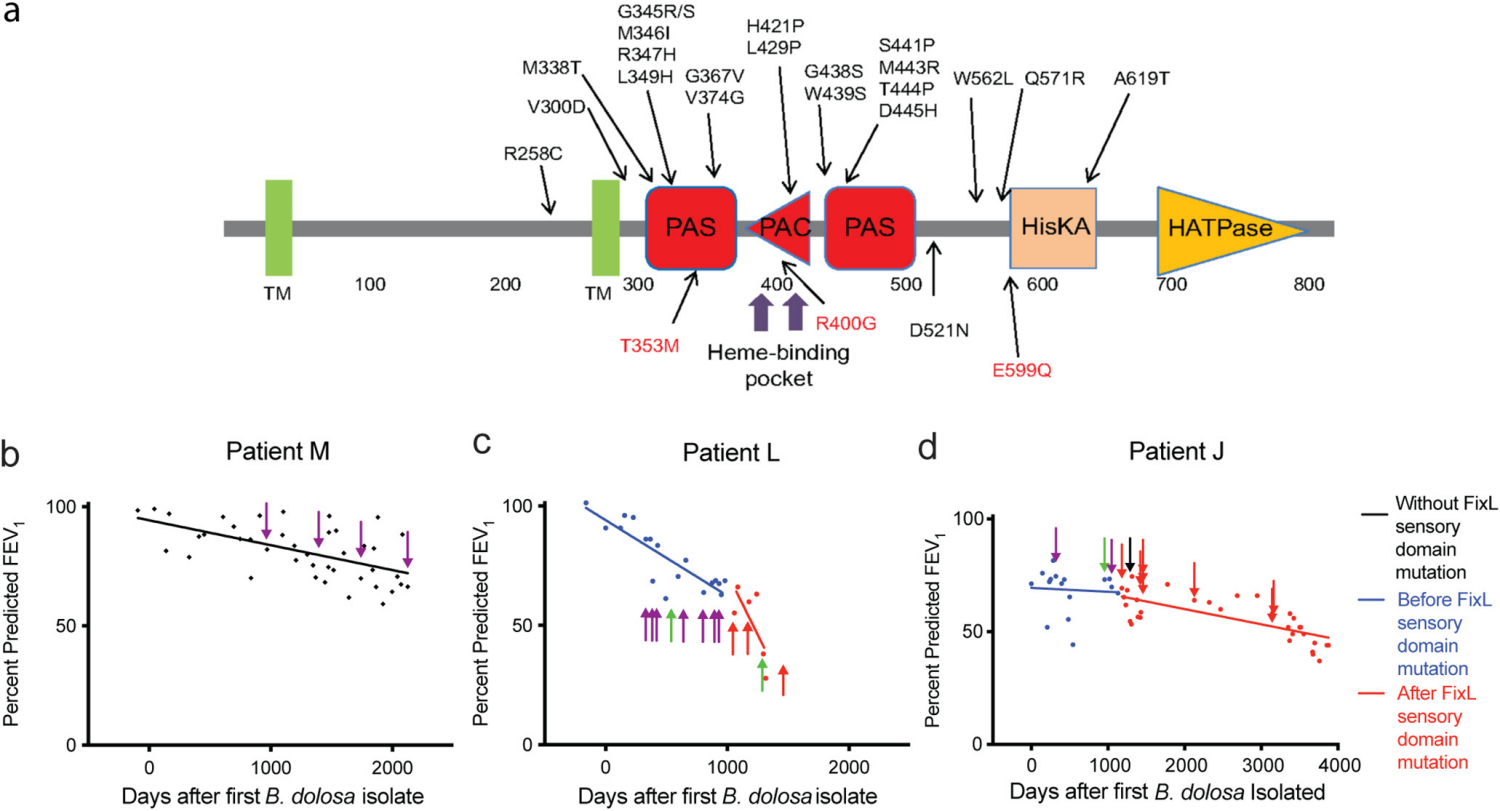
Mutations in the predicted sensory domain of FixL in *B. dolosa* are associated with decline in lung function in patients with cystic fibrosis. (a) Domains predicted by SMART (68) and mutations coded by observed single nucleotide polymorphisms (SNPs) in *B. dolosa* in black (17, 18) and *B. multivorans* in red (19). Domain abbreviations: TM, transmembrane; PAC, motif C-terminal to PAS motif; HisKA, histidine kinase; HATPase, histidine kinase-associated ATPase. (b) A modest decline was observed in percent predicted FEV1 (ppFEV1) in patient M who did not have a detectable *fixL* mutation. Rapid declines in ppFEV1 were seen in CF patients L (c) and J (d) after detection of mutations predicted to be in the sensory domain (PAS) of FixL; purple arrow, ancestral *fixL* allele; red arrow, *fixL* mutation in sensory domain; green arrow, *fixL* mutation not in sensory domain; black arrow, *fixJ* mutation. In c, a *P* value of 0.05 by linear regression comparing ppFEV1 slopes before and after detection of *fixL* mutations in the sensory domain is indicated by red arrows. In d, the slope was significantly different from 0 after detection of *fixL* mutations in the sensory domain (*P* < 0.0001 by linear regression), while the slope was not significantly different from 0 before detection of *fixL* mutations in the sensory domain.

In a series of published works involving more than 100 *B. dolosa* longitudinal isolates taken over 16 years from 14 different CF patients and 22 *B. multivorans* isolates taken from a single CF patient over 20 years, we identified mutations within *fixLJ* during chronic lung infection (17–19). The predicted domains of FixL along with the amino acid changes encoded by the clinically observed mutations are depicted in Fig. 1a. Most of the amino acid changes are within the predicted PAS and PAC domains, which are conserved sensory domains (23). Based on our previous work (21) and the predicted heme-binding pocket, it appears that the BCC *fixLJ* encodes an oxygen-sensing mechanism. Of note, the FixL proteins of *B. dolosa* (AK34_969) and *B. multivorans* (BMD20_10585) share 98% amino acid identity.

By retrospectively reviewing available clinical records from CF patients included in our previous *B. dolosa* whole-genome sequencing studies (17, 18), we compared the lung function of patients who were infected with *B. dolosa* isolates containing *fixL* mutations to determine if there was a correlation between *fixL* mutations and clinical outcomes. Of the 14 patients from our previous studies (17, 18), only 3 patients had sufficient lung function data that corresponded to the times when *B. dolosa* isolates were collected and sequenced. A steeper decline in lung function, measured by percent predicted forced expiratory volume in 1 s (ppFEV1), was seen in patient L after *B. dolosa* isolates with amino acid changes in the predicted sensory domain of FixL (red arrows) were detected. This slope differs in comparison to earlier time points, before such mutations were detected (Fig. 1c, purple and green arrows, red versus blue line; *P* = 0.05 comparing slopes using linear regression). The single green arrow among the purple arrows demonstrates that multiple lineages coexisted at the same time in the lung (17). Patient J (Fig. 1d) also had an increased rate of decline in lung function after *B. dolosa* isolates with amino acid changes in the predicted sensory domain were detected. Another patient, patient M (Fig. 1b), who lacked isolates containing mutations in *fixL*, had a more modest decline in lung function over a similar period. These findings suggest a correlation between evolved *fixL* variants and decreased lung function.

To determine the specific phenotypes of evolved *fixL* alleles, we generated otherwise isogenic mutant strains in reference isolate *B. dolosa* AU1058. The *fixLJ* deletion strain (21) was complemented with the ancestral *fixL* sequence or the evolved sequence found in strain AU0158 (encoding a FixL W439S amino acid change). It is worth pointing out that the reference strain AU0158 is itself an evolved strain as it was isolated ∼3 years after initial *B. dolosa* infection. Strains containing the *fixL* alleles that were associated with lower lung function in patient L (Fig. 1c) were also generated, encoding FixL G345S or R347H. These mutants were also complemented with the conserved ancestral sequence of *fixJ* along with ∼600 bp upstream, allowing for expression from its native promoter, using a mini-Tn7-based vector, allowing for long-term stability without the need for selection by the insertion into the chromosome (24). We also compared the effects that *fixL* sequence variants have on virulence in *B. multivorans* by generating otherwise isogenic mutants within *B. multivorans* VC7102 (BM2) by replacing a fragment of the *fixL* gene in its native location with a fragment with the desired evolved mutation (25). *B. multivorans* VC7102 was isolated from a patient with CF with a chronic BCC infection and later had isolates with *fixL* mutations (19). We found that the *B. dolosa* construct carrying the ancestral *fixL* genotype had a slight decrease in growth when grown *in vitro* at 37°C with ambient oxygen (Fig. S1 in the supplemental material) compared to *B. dolosa* constructs carrying evolved *fixL* genotypes. This decrease was only noticeable during log-phase growth, and by 24 h, there was only a slight, albeit statistically significant, growth defect. Consistent with our previous findings (21), *B. dolosa* lacking *fixLJ* had a greater defect in growth than *B. dolosa* carrying any *fixL* genotype. *B. multivorans* carrying ancestral *fixL* genotypes also had a slight growth defect during the log phase compared to *B. multivorans* carrying evolved *fixL* genotypes that recovered by 24 h of growth (Fig. S1).

10.1128/mBio.01823-21.1 FIG S1 *B. dolosa* and *B. multivorans* carrying the ancestral *fixL* allele have slight growth defects. *B. dolosa* (A) or *B. multivorans* (B) carrying *fixL* alleles grown in ambient oxygen at 37°C with agitation when OD_600_ was determined at the indicated time points. Data are representative of two separate experiments with three biological replicates per experiment; points are means, and error bars represent standard deviation (SD) and are not visible since they are smaller than the symbols. (A) *, *P* < 0.05 *ΔfixLJ* + FixLJ and *ΔfixLJ* + empty vector relative to any evolved variants; ^#^, *P* < 0.05 *ΔfixLJ* + FixLJ relative to *ΔfixLJ* + empty vector. (B) *, *P* < 0.05 VC7102 relative to VC7102 (FixL T353M) and VC7102 (FixL R400G); ^#^, *P* < 0.05 VC7102 relative to VC7102 (FixL T353M). An ANOVA with Sidak’s multiple-comparison test was used for all comparisons. Download FIG S1, TIF file, 0.2 MB. Copyright © 2021 Schaefers et al. 2021 Schaefers et al. https://creativecommons.org/licenses/by/4.0/ This content is distributed under the terms of the Creative Commons Attribution 4.0 International license.

### BCC strains carrying evolved *fixL* alleles are better able to survive within human macrophages and a murine pneumonia model but are less able to survive in soil.

**FIG 2:**
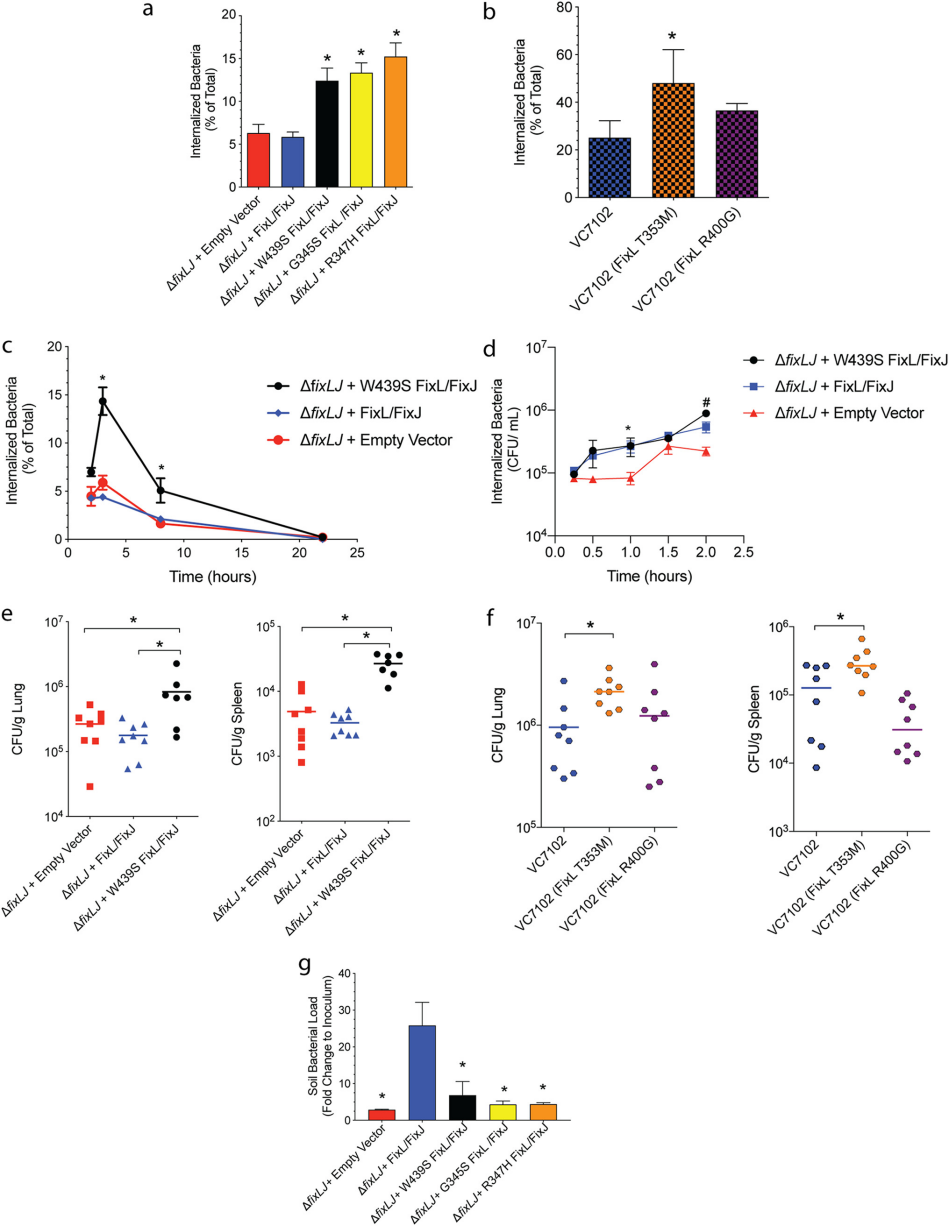
Burkholderia cepacia complex strains with evolved *fixL* alleles are more virulent in human macrophages and a murine pneumonia model but are less able to survive in soil. PMA-treated THP-1 human macrophages were infected with ∼2 × 10^6^ CFU/well (MOI of ∼10:1) of (a) *B. dolosa* strain AU0158 or (b) *B. multivorans* strain VC7102 otherwise isogenic mutants carrying different *fixL* alleles for 2 h, after which the percentage of internalized bacterial relative to the total bacterial growth was determined by killing extracellular bacteria with kanamycin (1 mg/ml). Means from 2 to 3 separate experiments with three replicates per experiment are plotted with error bars representing one standard deviation; *, *P* < 0.05 by analysis of variance (ANOVA) with Tukey’s multiple-comparison test compared to (a) Δ*fixLJ +* empty vector and Δ*fixLJ +* FixL/FixJ or (b) strain VC7102. (c) THP-1-derived macrophages were infected with ∼2 × 10^6^ CFU/well of *B*. *dolosa* for 2 h, after which the extracellular bacteria were treated with kanamycin (1 mg/ml) for various amounts of time. The, the percentage of internalized bacteria relative to the total bacterial growth within the initial 2-h infection was determined; *, *P* < 0.05 by ANOVA with Tukey’s multiple-comparison test compared to Δ*fixLJ +* FixL/FixJ. (d) THP-1-derived macrophages were infected with ∼2 × 10^6^ CFU/well of *B*. *dolosa* for various amounts of time (15 min to 2 h), after which the number of internalized bacteria was determined by killing extracellular bacteria with kanamycin (1 mg/ml); *, *P* < 0.05 by ANOVA with Tukey’s multiple-comparison test compared to Δ*fixLJ +* empty vector; ^#^, *P* < 0.05 by ANOVA with Tukey’s multiple-comparison test compared to Δ*fixLJ +* FixL/FixJ. C57BL/6 mice were intranasally challenged with ∼4 × 10^8^ CFU/mouse of (e) *B. dolosa* strain AU0158 or (f) *B. multivorans* strain VC7102 mutants carrying *fixL* alleles. Bacterial loads were measured in the lungs and spleen 7 days after infection. Data are representative of two separate experiments with 7 to 8 mice per group. Each point represents one mouse, and bars represent medians; *, *P* < 0.05 by ANOVA with Tukey’s multiple-comparison test. (g) *B. dolosa* strain AU0158 Δ*fixLJ* mutants complemented with *fixL* alleles or empty vector were inoculated into 1 g of sterile soil (1 to 6 × 10^6^ CFU in minimal medium) and incubated for 10 days. Bacterial load was measured and plotted relative to the inoculum used; *, *P* < 0.05 compared to Δ*fixLJ +* FixL/FixJ by ANOVA with Tukey’s multiple-comparison test.

We compared the virulence of the BCC *fixL* mutants by measuring their ability to invade/survive within macrophages and within the lungs and spleen in a murine pneumonia model. To measure bacterial invasion/survival within macrophages, we used THP-1 human monocyte cells treated with phorbol 12-myristate 13-acetate (PMA) to differentiate them into macrophage-like cells. *B. dolosa* mutants carrying the evolved *fixL* allele (encoding FixL W439S) or the evolved *fixL* alleles associated with a period of clinical decline (encoding FixL G345S or R347H) were detected at significantly increased levels (2- to 3-fold higher) compared to mutants carrying the ancestral *fixL* sequence or lacking *fixLJ* (empty vector) (Fig. 2a). One of the two *B. multivorans* mutants carrying the evolved *fixL* allele (encoding FixL T353M) had increased bacterial loads within THP-1-dervived macrophages compared to the ancestral strain (VC7102) (Fig. 2b). This increase in bacterial load within the THP-1-dervived macrophages could be a function of either increased uptake of the bacteria or increased survival within the macrophage. Our previous work found that *B. dolosa* lacking *fixLJ* was equally able to be taken up by macrophages while it was less able to survive within macrophages than *B. dolosa* containing *fixLJ* (21). To determine the mechanism of the increased bacterial load, we conducted a series of time course experiments where we varied the length of the infection before the addition of kanamycin (which kills the extracellular bacteria) to determine the uptake/invasion of the bacteria by the macrophages. We also varied the length of kanamycin exposure to determine the ability of the bacteria to survive once intracellular. We found that the *B. dolosa* mutant carrying the evolved *fixL* allele (encoding FixL W439S) had significantly increased survival (increased bacterial load) within macrophages at 4 and 8 h after a 2-h infection compared to the strain with the *B. dolosa* ancestral *fixL* genotype or the strain lacking *fixLJ* (Fig. 2c). We also compared the uptake of the *B. dolosa fixL* mutants by THP-1-derived macrophages (Fig. 2d) and found that all the *fixL* mutants had equal uptake at initial time points. *B. dolosa* carrying the evolved *fixL* allele (encoding FixL W439S) had an increased number of intracellular bacteria at the 2-h time point, which was likely due to its increased ability to survive within the macrophages. These data demonstrate that *B. dolosa* strains carrying evolved *fixL* alleles are better able to survive within macrophages than strains carrying the ancestral *fixL* allele, making these strains carrying evolved *fixL* alleles more virulent.

Our previous work found a correlation between the ability to survive within THP-1-dervived macrophages and the ability to persist within murine lungs and to disseminate and persist in the spleen after intranasal inoculation (21). In order to measure the ability of the *fixL* mutants to persist *in vivo*, C57BL/6 mice were intranasally infected with these BCC mutants using similar methods. Mice infected with *B. dolosa* carrying the evolved *fixL* allele (encoding FixL W439S) had 4- to 5-fold higher levels of bacteria within the lungs and spleen than mice infected with *B. dolosa* carrying the ancestral *fixL* allele or lacking *fixLJ* (empty vector) (Fig. 2e), indicating that these strains carrying evolved *fixL* alleles are more virulent. Mice that were infected with *B. multivorans* VC7102 carrying the evolved *fixL* allele (encoding FixL T353M) had significantly increased bacterial loads in the lungs and spleen compared to mice infected with bacteria carrying the ancestral *fixL* allele, while the other evolved *fixL* mutation (encoding FixL R400G) did not (Fig. 2f). The FixL T353M-expressing *B. multivorans* mutant also was better able to survive within the THP-1-derived macrophages than the ancestral strain, while the FixL R400G-expressing *B. multivorans* mutant was not (Fig. 2b). It is likely that the mutation encoding the R400G mutation in FixL confers a benefit that was not investigated in this study.

To determine if the *fixL* mutations that confer an increased level of virulence within the host came at the expense of survival in the soil, a natural ecological niche of the BCC, we measured the ability of the *B. dolosa fixL* mutants to survive within soil for 10 days. We found that all three of the strains carrying evolved *fixL* alleles (encoding FixL W439S, G345S, or R347H) had an ∼80 to 85% reduction in the ability to survive within soil compared to the strain carrying the ancestral *fixL* allele (Fig. 2g). Bacteria lacking *fixLJ* had reduced survival in the soil, demonstrating the importance of the *fixL* gene to survival both within the host and in the environment.

### BCC strains carrying evolved *fixL* alleles are more motile, make less biofilm, and have altered gene expression.

**FIG 3:**
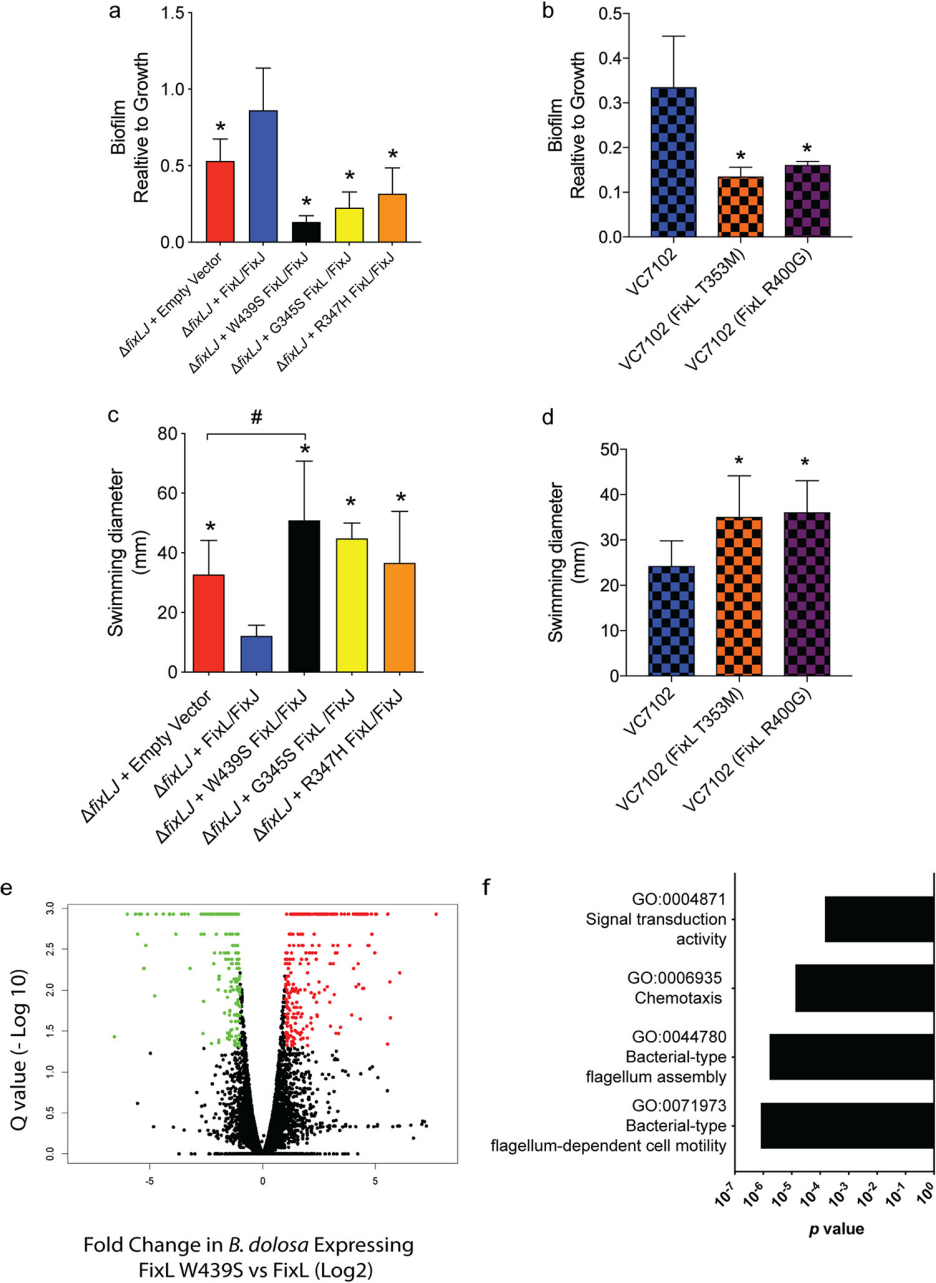
Burkholderia cepacia complex strains with evolved *fixL* alleles were more motile, made less biofilm, and had altered gene expression. Biofilm formation of (a) *B. dolosa* strain AU0158 or (b) *B. multivorans* strain VC7102 mutants carrying *fixL* alleles on PVC plates as measured by crystal violet staining at 48 h. Means from three separate experiments with 5 to 6 replicates per experiment are plotted with error bars representing one standard deviation; *, *P* < 0.05 by ANOVA with Tukey’s multiple-comparison test to Δ*fixLJ+* FixL/FixJ or VC7102. Motility of (c) *B. dolosa* strain AU0158 or (d) *B. multivorans* strain VC7102 mutants carrying *fixL* alleles on low-density (0.3%) LB agar and swimming distance were measured after incubation for 48 h. Means from three separate experiments with 3 to 4 replicates per experiment are plotted with error bars representing one standard deviation. *, *P* < 0.05 by ANOVA with Tukey’s multiple-comparison test compared to construct carrying ancestral *fixL* allele; ^#^, *P* < 0.05 by ANOVA with Tukey’s multiple-comparison test. (e) Volcano plot depicting the differential regulation of genes. Green dots signify genes with expression 2-fold lower in the *B. dolosa* strain AU0158 mutant carrying the evolved *fixL* allele (encoding FixL W439S) than in a mutant carrying the ancestral *fixL* allele, with a *q* value of <0.05. Red dots signify genes with expression 2-fold higher in the mutant carrying the evolved *fixL* allele (encoding FixL W439S) than in a mutant carrying the ancestral *fixL* allele, with a *q* value of <0.05. (f) GO terms that were enriched with an adjusted *P* value of <0.05 among genes that were statistically upregulated (*q* value of <0.05, at least 2-fold) in *B. dolosa* carrying the evolved *fixL* allele (encoding FixL W439S) relative to *B. dolosa* carrying the ancestral *fixL* allele.

Our previous work showed that *B. dolosa* lacking *fixLJ* produced increased levels of biofilm and had decreased motility compared to *B. dolosa* containing *fixLJ* (21). We found that both *B. dolosa* and *B. multivorans* carrying evolved *fixL* alleles produced significantly less biofilm than otherwise isogenic strains carrying the ancestral *fixL* sequence (Fig. 3a and b), although overall biofilm production in this assay was low. To further evaluate the potential contribution of biofilm in the *in vitro* invasion assay described above, we performed Wright-Giemsa staining of THP-derived macrophages infected with *B. dolosa*. We observed that *B. dolosa* carrying either the ancestral *fixL* sequence or an evolved *fixL* sequence (encoding FixL W439S) was not seen in biofilm-like clusters but was instead observed to exist as single or small clusters made up of no more than 4 cells, with ∼80% of the aggregates consisting of 1 to 2 bacterial cells (Fig. S2). *B. dolosa* and *B. multivorans* mutants with evolved *fixL* alleles also had increased motility compared to mutants carrying the ancestral *fixL* allele (Fig. 3c and d). Interestingly, *B. dolosa* carrying the ancestral *fixL* allele was completely nonmotile, where the diameter plotted in Fig. 3c (∼12 mm) was the diameter of the 10-μl drop placed on the agar surface. *B. dolosa* carrying an empty vector, which lacks *fixLJ*, still had the ability to swim, albeit at lower levels than *B. dolosa* carrying an evolved *fixL* allele (Fig. 3c).

10.1128/mBio.01823-21.2 FIG S2 *B. dolosa* carrying evolved and ancestral *fixL* alleles do not form biofilm-like clusters when infecting THP-1-derived macrophages. THP-1-derived macrophages were infected with ∼2 × 10^5^ CFU *B. dolosa* either carrying an evolved *fixL* allele (encoding FixL W439S) (a) or the ancestral *fixL* sequence (b) for 2 h. Infected cells were then rinsed with PBS and stained with a Hema-3 stain set and visualized using light microscopy. The white bar denotes 10 μm. (c) the number of bacterial cells in 20 to 70 aggregates per slide was quantified and plotted as the percentage of aggregates with the indicated number of bacteria. Three to four slides per strain (three to six fields per slide) were counted, and the median with interquartile range is plotted. Download FIG S2, PDF file, 0.4 MB. Copyright © 2021 Schaefers et al. 2021 Schaefers et al. https://creativecommons.org/licenses/by/4.0/ This content is distributed under the terms of the Creative Commons Attribution 4.0 International license.

To identify the differentially expressed genes that were responsible for the various observed phenotypes, we measured global transcript levels using RNA sequencing (RNA-seq) of bacteria grown *in vitro* to log phase (Table S1). Previously we found that ∼11% of the genome was differentially expressed in a *B. dolosa fixLJ* deletion mutant compared to the parental strain (21). Here, the *B. dolosa* FixL W439S-expressing mutant had 205 genes that were significantly downregulated and 302 genes that were significantly upregulated (*q* value of <0.05, at least 2-fold difference; Fig. 3e) compared to *B. dolosa* carrying the ancestral *fixL* allele. We sought to identify pathways that were differentially regulated by analyzing enriched Gene Ontology (GO) terms of the genes that were significantly differentially expressed. Surprisingly, there were no GO terms that were enriched among genes that were significantly upregulated in *B. dolosa* carrying the ancestral *fixL* allele compared to *B. dolosa* carrying the evolved *fixL* allele. However, there were four GO terms that were enriched among genes that were differentially upregulated in *B. dolosa* carrying the evolved *fixL* allele compared to *B. dolosa* carrying the ancestral *fixL* allele (encoding FixL W439S; Fig. 3f). Three of these GO terms are associated with motility or chemotaxis and the fourth is associated with signal transduction. A subset of the significantly upregulated genes associated with motility and flagellar assembly in the *B. dolosa* FixL W439S-expressing mutant are listed in Table 1. Additionally, we confirmed the differential expression levels of *fliC* and *motA* transcripts in *B. dolosa* carrying the ancestral *fixL* allele, evolved *fixL* allele (encoding FixL W439S), and empty vectors using reverse transcription-quantitative PCR (qRT-PCR) (Fig. S3).

**TABLE 1:** Selected genes associated with motility and/or flagellar assembly that are significantly differentially expressed in *B. dolosa* carrying the evolved *fixL* allele (encoding FixL W439S) relative to *B. dolosa* carrying the *fixL* ancestral allele

Gene name (genome designation)	Description	Fold change in *B. dolosa* carrying evolved *fixL* allele (encoding FixL W439S)
AK34_2913	Flagellin protein FliC	205.8
AK34_2901	Flagellar motor rotation protein MotA	46.8
AK34_2900	Flagellar motor rotation protein MotB	19.9
AK34_2902	Flagellar transcriptional activator FlhC	24.9
AK34_2903	Flagellar transcriptional activator FlhD	10.9
AK34_2914	Flagellar cap protein FliD	22.7
AK34_2885	Flagellar biosynthesis protein FlhA	46.5
AK34_2886	Flagellar biosynthesis protein FlhB	28.1
AK34_83	Flagellar basal-body P-ring formation protein FlgA	18.2
AK34_86	Flagellar basal-body rod modification protein FlgD	49.8
AK34_87	Flagellar hook protein FlgE	32.0
AK34_88	Flagellar basal-body rod protein FlgF	50.5
AK34_2914	Flagellar cap protein FliD	22.7
AK34_3043	Flagellar motor switch protein FliM	17.2
AK34_3044	Flagellar motor switch protein FliN	9.0

10.1128/mBio.01823-21.3 FIG S3 *B. dolosa* carrying an evolved *fixL* allele has higher levels of *fliC* and *motA* mRNA than *B. dolosa* carrying an ancestral *fixL* allele. Relative levels of *fliC* (A, C) and *motA* (B, D) in the *B. dolosa fixLJ* deletion mutant complemented with the ancestral *fixL* allele, evolved FixL allele (encoding FixL W439S), or empty vector measured by qRT-PCR normalized to the levels of *rpoD* (A, B) or *gyrB* (C, D). Plots are representative of two separate experiments with two to three biological replicates per experiment; error bars represent SD; *, *P* < 0.05 by ANOVA with Tukey’s multiple-comparison test FIG S3, TIF file, 0.4 MB. Copyright © 2021 Schaefers et al. 2021 Schaefers et al. https://creativecommons.org/licenses/by/4.0/ This content is distributed under the terms of the Creative Commons Attribution 4.0 International license.

10.1128/mBio.01823-21.7 TABLE S1 Transcript levels and fold change in AU0158 *fixLJ* deletion mutant + FixLJ (ancestral) compared to *B. dolosa* AU0158 *fixLJ* deletion mutant + W439S FixLJ (evolved) measured by RNA-seq. Download Table S1, XLSX file, 0.5 MB. Copyright © 2021 Schaefers et al. 2021 Schaefers et al. https://creativecommons.org/licenses/by/4.0/ This content is distributed under the terms of the Creative Commons Attribution 4.0 International license.

We also identified several differentially expressed genes with homologs in other bacteria that have been shown to play a role in cyclic diguanylate monophosphate (c-di-GMP) metabolism that are important for motility and biofilm regulation (Table S2). Notably, a homolog of the predicted phophodiesterase *cpdA* that could hydrolyze c-di-GMP, AK34_1958, was significantly downregulated in *B. dolosa* carrying the ancestral *fixL* allele compared to in *B. dolosa* carrying the evolved *fixL* allele (∼8.5-fold; Table S2). B. pseudomallei (26) and B. cenocepacia (27, 28) mutants lacking *cpdA* had reduced motility, consistent with the *B. dolosa* strain carrying the ancestral *fixL* allele, which had lower *cpdA* transcript levels and lower motility than *B. dolosa* carrying the evolved *fixL* allele. Similar to B. pseudomallei and B. cenocepacia *cpdA*, *B. dolosa* AU0158 *cpdA* has predicted GGDEF and EAL domains, but only the EAL domain responsible for phosphodiesterase activity is predicted to be enzymatically active based on amino acid sequence at the catalytic site. B. pseudomallei (26) and B. cenocepacia (27) mutants lacking *cpdA* had increased levels c-di-GMP. The expression changes of these genes (Table S2) suggest that decreased intracellular c-di-GMP levels may be found in the *B. dolosa* mutants carrying evolved *fixL* alleles, and this may explain the increased motility and decreased biofilm seen in these mutants (26, 28, 29). But, surprisingly, there was a significant increase in intracellular c-di-GMP levels found in the *B. dolosa* mutant carrying the evolved *fixL* allele (Fig. S4).

10.1128/mBio.01823-21.4 FIG S4 *B. dolosa* carrying an evolved *fixL* allele has higher levels of c-di-GMP. c-di-GMP was extracted from stationary-phase *B. dolosa* and was measured by mass spectrometry. Mean data from three biological replicates are shown. Error bars represent standard deviation. *, *P* < 0.05 by one-way ANOVA with Tukey’s multiple-comparison test compared to *ΔfixLJ* + FixLJ or *ΔfixLJ* + empty vector. Download FIG S4, TIF file, 0.09 MB. Copyright © 2021 Schaefers et al. 2021 Schaefers et al. https://creativecommons.org/licenses/by/4.0/ This content is distributed under the terms of the Creative Commons Attribution 4.0 International license.

10.1128/mBio.01823-21.8 TABLE S2 Selected genes of interest that are significantly differentially expressed in *B. dolosa* carrying the ancestral *fixL* allele relative to *B. dolosa* carrying the evolved *fixL* allele. Download Table S2, DOCX file, 0.01 MB. Copyright © 2021 Schaefers et al. 2021 Schaefers et al. https://creativecommons.org/licenses/by/4.0/ This content is distributed under the terms of the Creative Commons Attribution 4.0 International license.

### Evolved *fixL* alleles downregulate *fix* pathway activity.

**FIG 4:**
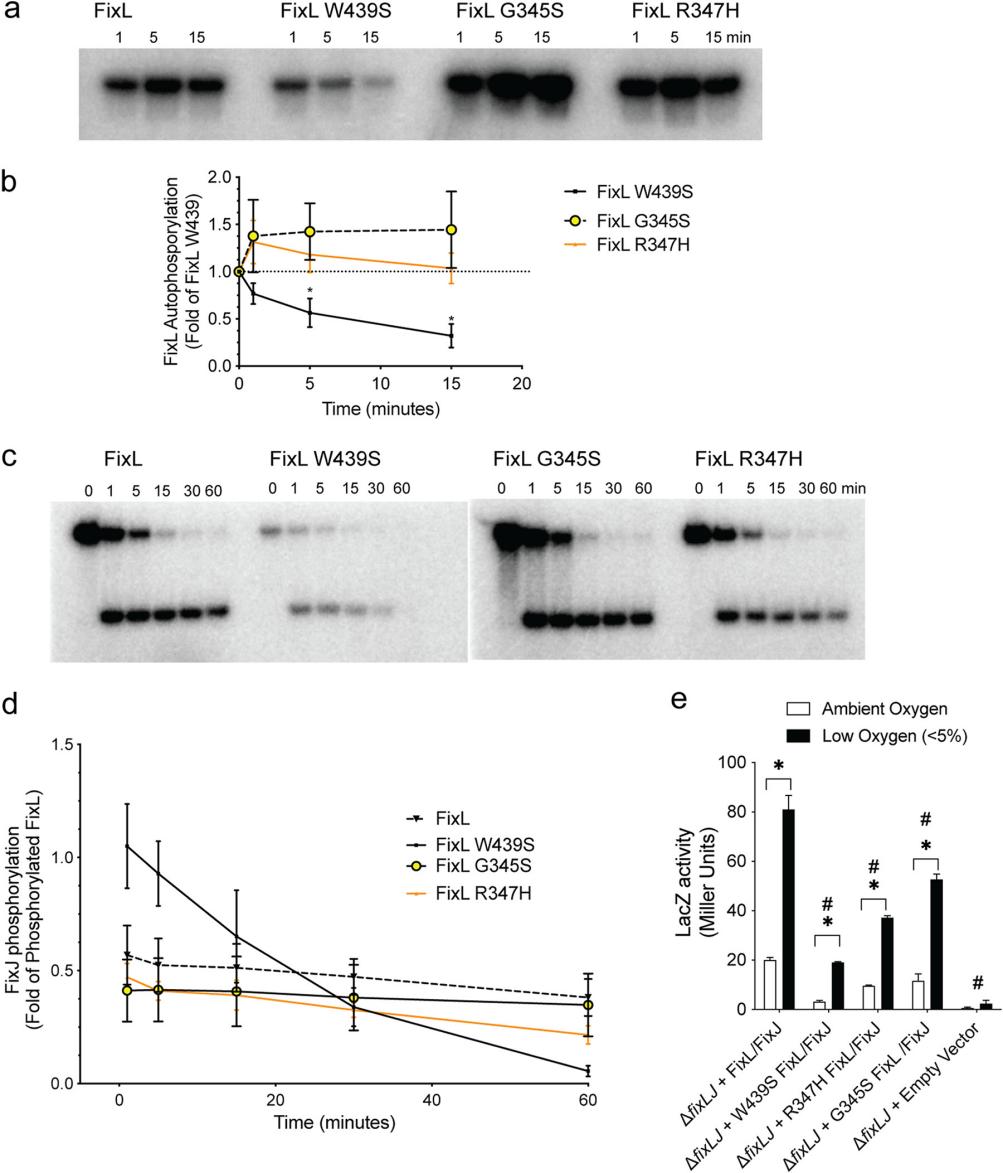
Evolved *fixL* alleles downregulate *fix* pathway activity. (a) Representative plots of autophosphorylation of *B. dolosa* FixL proteins. (b) Density measurements from three independent experiments were normalized relative to FixL at the same time point. (c) Representative plots of phosphotransfer to FixJ of *B. dolosa* FixL proteins. (d) Density measurements from three independent experiments were normalized based on the level of FixL phosphorylation at time zero for each construct. (e) *B. dolosa* strain AU0158 mutants carrying *fixL* alleles or an empty vector carrying a *pfixK-lacZ* reporter plasmid (21) grown in ambient or low (<5%) oxygen conditions. Bars represent the means of triplicate biological replicates, and error bars represent one standard deviation (representative of three independent experiments); *, *P* < 0.05 by ANOVA with Tukey’s multiple-comparison test for growth in ambient oxygen compared to growth under low oxygen; ^#^, *P* < 0.05 by ANOVA with Tukey’s multiple-comparison test to Δ*fixLJ+* FixL/FixJ in both ambient and low oxygen.

Most two-component systems function by regulating gene expression when activated by a specific signal. The first component, the sensor kinase, senses the signal and autophosphorylates. The phosphate is then transferred to the second component, the response regulator, which can then regulate gene transcription (20). We conducted *in vitro* phosphorylation assays with recombinant truncated FixL and full-length FixJ proteins to measure the ability of the different FixL variants to phosphorylate themselves and then subsequently FixJ. The recombinant FixL was truncated to exclude the predicted transmembrane domains to facilitate protein purification, so the recombinant protein began at amino acid 329. The recombinant FixL proteins still retain the predicted PAS/PAC sensory domain (with oxygen-binding heme moiety) and histidine kinase domain. The use of similar truncated recombinant proteins in *in vitro* phosphotransfer experiments has been reported for other two-component systems (30–32). We hypothesized that FixL variants that had different phenotypes would have differing levels of autophosphorylation and/or phosphotransfer. Figure 4a shows that the ancestral FixL had higher levels of autophosphorylation than the evolved FixL W439S. When the levels of autophosphorylation were quantified, the evolved FixL W439S had approximately 50% of the level of autophosphorylation of the ancestral sequence (Fig. 4b). Interestingly, the other two evolved FixL proteins, G345S and R347H, had increased levels of autophosphorylation compared to the ancestral protein (Fig. 4a and b) despite making less biofilm and being more motile like the other evolved protein FixL W439S. Equal amounts of each of the purified protein were used in each reaction, and each protein preparation was >90% pure (Fig. S5). When the autophosphorylation levels were quantified relative to the 1-min time point, only the FixL W439S autophosphorylation levels decreased, while the other three variants increased or stayed the same (Fig. S6). We also measured the ability of the recombinant FixL proteins to phosphorylate the response regulator FixJ. As the evolved FixL W439S protein had lower levels of autophosphorylated FixL, that variant had lower levels of phosphotransfer to FixJ than the ancestral protein (Fig. 4c and d). The evolved FixL W439S protein had a different phosphotransfer profile compared to the ancestral protein and the other two evolved proteins when the level of phosphorylated FixJ was quantified relative to the level of autophosphorylated FixL. The FixL W439S protein had a more rapid phosphotransfer to FixJ and potentially a greater level of dephosphorylation of FixJ (Fig. 4d). The other two evolved FixL proteins R347H and G345S had similar levels of phosphotransfer to ancestral FixL (Fig. 4d) despite having an increased ability to autophosphorylate compared to ancestral FixL (Fig. 4b). To further explore and quantify the effects of *fixL* mutations on the *fix* pathway in *B. dolosa*, we also measured *fix* activity using a LacZ reporter conjugated into *B. dolosa* carrying the various *fixL* alleles (21). We found that *B. dolosa* carrying the ancestral and the evolved *fixL* alleles (encoding FixL W439S, G345S, or R347H) had an increase in *fix* pathway activity when the construct was grown in low oxygen (<5%), demonstrating that all alleles are activated in low oxygen (Fig. 4e). Consistent with the *in vitro* phosphorylation experiments, *B. dolosa* carrying the ancestral *fixL* sequence had higher *fix* pathway activity than *B. dolosa* carrying the evolved *fixL* allele (encoding FixL W439S) when grown in either ambient or low oxygen. *B. dolosa* carrying the other two evolved *fixL* alleles (encoding FixL G345S or R347H) had lower *fix* pathway activity than *B. dolosa* carrying the ancestral allele in both ambient and low oxygen, demonstrating decreased *fix* pathway activity in all the *B. dolosa* mutants carrying evolved *fixL* alleles.

10.1128/mBio.01823-21.5 FIG S5 *B. dolosa* FixL and FixJ protein preparations. Various FixL proteins (1.2 μg) or FixJ (1 μg) was run on an SDS-PAGE gel and stained with Coomassie stain. Protein ladder sizes in kDa are listed. The expected size of FixL is ∼115 kDa and FixJ is ∼40 kDa. Download FIG S5, PDF file, 0.2 MB. Copyright © 2021 Schaefers et al. 2021 Schaefers et al. https://creativecommons.org/licenses/by/4.0/ This content is distributed under the terms of the Creative Commons Attribution 4.0 International license.

10.1128/mBio.01823-21.6 FIG S6 W439S FixL has lower autophosphorylation activity than ancestral FixL. Data from Fig. 4B were normalized to the 1-min time point for each variant. *, *P* < 0.05 compared to FixL at time point by ANOVA with Tukey’s multiple-comparison test. Download FIG S6, TIF file, 0.1 MB. Copyright © 2021 Schaefers et al. 2021 Schaefers et al. https://creativecommons.org/licenses/by/4.0/ This content is distributed under the terms of the Creative Commons Attribution 4.0 International license.

## DISCUSSION

In previous work, we demonstrated that the BCC two-component system encoded by *fixLJ* shows evidence of positive selective pressure during chronic lung infection in CF patients (17–19) and is critical for full BCC virulence (21). In this study, we evaluated the function of evolved *fixL* sequence variations in both *B. dolosa* and *B. multivorans* by generating otherwise isogenic mutants carrying the evolved *fixL* alleles. These mutants carrying evolved *fixL* alleles were more virulent (as having increased bacterial levels within tissue or macrophages), more motile, and produced less biofilm (Fig. 2 and 3). The *fixL* mutations that occurred during chronic infection downregulated *fix* pathway signaling, demonstrating that high *fix* pathway activity is associated with lowered virulence (Fig. 4).

We found that BCC carrying ancestral *fixL* sequences had reduced motility and reduced virulence in both murine and macrophage models compared to variants carrying evolved sequences (Fig. 2 and 3), suggesting that reduced motility was contributing to the reduced virulence, as has been suggested by other studies (33–35). Our previous work demonstrated that *B. dolosa* lacking flagella were equally virulent as parental flagellated strains, suggesting that motility plays a minimal role in the infection models evaluated (21). We analyzed the transcriptomes of *B. dolosa* mutants carrying ancestral or evolved *fixL* alleles to understand the mechanisms of the altered phenotypes seen between the two mutants. Among the genes that were identified to be differentially expressed between *B. dolosa* carrying different *fixL* sequences were genes that have homologs in other bacterial species that are involved in c-di-GMP metabolism (Table S2 in the supplemental material) (26, 28, 29). Surprisingly, *B. dolosa* carrying the ancestral *fixL* sequence that produced more biofilm had lower levels of c-di-GMP than *B. dolosa* carrying the evolved *fixL* allele (Fig. S3). The increased biofilm seen in *B. dolosa* carrying the ancestral *fixL* allele and in *B. dolosa* lacking *fixLJ* was independent of increased c-di-GMP levels, indicating that there are additional pathways involved in biofilm formation. Furthermore, high levels of c-di-GMP have been shown to stimulate the production of extracellular polysaccharides that leads to increased biofilm formation (28, 36). Interestingly, the genes that are responsible for c-di-GMP-induced production of polysaccharides (*Burkholderia cenocepacia* exopolysaccharide [Bep], poly-*N*-acetylglucosamine [PNAG], and cepacian) (37–39) were not differentially expressed by RNA-seq analysis (Table S1), suggesting that c-di-GMP-independent mechanisms of biofilm production are involved.

One potential c-di-GMP-independent mechanism of biofilm production involves the *wsp* system that can promote biofilm formation in B. cenocepacia without direct activation of a diguanylate cyclase (40, 41). Multiple components of the *wsp* system were 2- to 4-fold upregulated in *B. dolosa* carrying the ancestral *fixL* allele (Table S2), suggesting a potential role of the *wsp* system in *fix* pathway-mediated biofilm formation. The contribution of biofilm formation to BCC virulence remains unclear, as mutants carrying ancestral *fixL* sequences produced more biofilm and were less pathogenic in our murine model of pneumonia than isogenic constructs with evolved *fixL* alleles (Fig. 2 and 3). Similarly, in our previous study, *B. dolosa fixLJ* deletion mutants made more biofilm and were less pathogenic than the parental strain that contained *fixLJ* (21). These findings suggesting that BCC biofilms may not be beneficial for infection are supported by a study of CF lung explants from patients infected with Pseudomonas aeruginosa and/or BCC that were stained using species-specific antibodies (42). BCC bacteria were rarely found in biofilm-like structures, while P. aeruginosa were often found in such structures, suggesting that BCC may not form large biofilms during infection but may form small aggregates. The conversion of BCC from a mucoid, biofilm-producing state to a nonmucoid phenotype is often observed during chronic infection and is correlated with worse clinical outcomes (notably different from P. aeruginosa, where mucoid strains are associated with clinical decline) (43). Interestingly, these more virulent isolates are found early during infection for P. aeruginosa but are found later during infection for CF patients infected with BCC (43, 44).

Since increased biofilm formation is not associated with increased BCC virulence (Fig. 2 and 3), we hypothesized that biofilm formation may allow for *B. dolosa* to better survive within the soil. But, the ability to make biofilms does not, on its own, confer the ability to survive within soil, as *B. dolosa* lacking *fixLJ* was unable to persist in the soil at high levels (Fig. 2) despite making higher levels of biofilm (Fig. 3). The mutations in *fixLJ* that confer increased virulence are likely dead-end mutations, as they make the bacteria less able to survive in the natural BCC reservoir. It is possible that these mutations make the bacteria more transmissible between human hosts; future work will investigate this.

To better understand the mechanism of altered phenotypes seen in the mutants carrying different *fixL* alleles, we examined genes that had the largest magnitude of differential expression observed by RNA-seq. One such gene was an AraC family transcription regulator (AK34_4608) that was significantly downregulated (∼95-fold) in *B. dolosa* carrying the evolved *fixL* allele (Table S2). Expression of this gene is potentially detrimental to bacterial dissemination since a homolog of this gene was found to be downregulated in B. cenocepacia blood isolates taken from CF patients with cepacia syndrome compared to in lung isolates taken at same time (45). Additionally, a homolog of this same gene was found to be downregulated in B. pseudomallei isolates from CF patients taken at late versus early time points (46). Other genes that were differentially expressed encode a putative CidA/CidB-like holin/antiholin system (AK34_3040, AK34_3041) that was significantly downregulated (∼64- and 42-fold, respectively) in *B. dolosa* carrying the evolved *fixL* allele (Table S2). Homologs of this system were found to upregulated in P. aeruginosa when grown *in vitro* with Staphylococcus aureus (47), suggesting that upregulation of this system may result in more autolysis and increased biofilm formation (48). Further investigation is needed to determine the role of these genes in BCC virulence and their role in *fix* pathway-mediated phenotypes.

In this study, we have characterized the effects of mutations within the oxygen-sensing two-component system encoded by *fixLJ* that arise during chronic infection in people with CF. Some of these mutations were associated with a period of clinical decline. Host-evolved BCC carrying *fixL* mutants were more virulent, more motile, and produced less biofilm. In contrast, P. aeruginosa becomes less motile, produces more biofilm, shows increased antibiotic resistance, and has increased auxotrophy in chronic infections, ultimately leading to the evolution of reduced virulence of late isolates in animal models of infection (49, 50). Less is known about phenotypic changes that occur during chronic BCC infection, and most prior work has focused on B. cenocepacia (51). The findings from this study demonstrate a novel way that the BCC adapts to the host by making the bacteria more pathogenic at the cost of being less able to survive within the environment.

## MATERIALS AND METHODS

### Clinical data.

Records of *B. dolosa-*infected patients were reviewed under Boston Children’s Hospital institutional review board protocol number M10-08-0370.

### Bacterial strains, plasmids, cell lines, and growth conditions.

All strains used and generated in this study are listed in Table 2. *B. dolosa* strain AU0158 was obtained from John LiPuma (University of Michigan) and is an early isolate from the index patient from the *B. dolosa* outbreak (about 3 years into the outbreak). BCC and E. coli were grown on LB plates or in LB medium and were supplemented with the following additives: ampicillin (100 μg/ml), kanamycin (50 μg/ml for E. coli, 1 mg/ml for BCC), trimethoprim (100 μg/ml for E. coli, 1 mg/ml for BCC), gentamicin (15 or 50 μg/ml), chloramphenicol (20 μg/ml), or diaminopimelic acid (200 μg/ml). Plasmids that were used in this study are listed in Table 3. The human monocyte line THP-1 was obtained from ATCC and grown at 37°C with 5% CO_2_. THP-1 cells were cultured in RPMI 1640 medium containing 2 mM l-glutamine, 10 mM HEPES, 1 mM sodium pyruvate, 4,500 mg/liter glucose, and 1,500 mg/liter sodium bicarbonate supplemented with 10% heat-inactivated fetal calf serum (FCS) (Invitrogen) and 0.05 mM 2-mercaptoethanol. Low-oxygen environments were generated using the CampyGen gas generating system (Thermo Fisher), and the low-oxygen concentration (<5%) is based on the manufacturer’s specifications.

**TABLE 2:** Strains used in this study

	Notes^*a*^	Source
E. coli		
NEB 5-alpha competent E. coli	DH5α derivative cloning strain	New England BioLabs
RHO3	Mobilizer strain. Km^s^; SM10(λ*pir*) Δ*asd*::*FRT* Δ*aphA*::*FRT*	25
BL21(DE3)	Protein expression strain	Invitrogen
BCC		
*B. dolosa* AU0158	Clinical isolate	John LiPuma
*B. multivorans* VC7102	Clinical isolate	19
Δ*fixLJ* + W439S *fixLJ*	*B. dolosa* Δ*fixLJ* + p*fixLJ* carrying W439S FixL and FixJ, integrated at *att*Tn7 site downstream of AK34_4894	21
Δ*fixLJ* + empty vector	*B. dolosa* Δ*fixLJ* + empty pUC18T-mini-Tn*7*T-Tp integrated at *att*Tn7 site downstream of AK34_4894	21
Δ*fixLJ* + *fixLJ*	*B. dolosa* Δ*fixLJ* + p*fixLJ* carrying W439 FixL and FixJ, integrated at *att*Tn7 site downstream of AK34_4894	This study
Δ*fixLJ* + R347H *fixLJ*	*B. dolosa* Δ*fixLJ* + p*fixLJ* carrying R347H FixL and FixJ, integrated at *att*Tn7 site downstream of AK34_4894	This study
Δ*fixLJ* + G345S *fixLJ*	*B. dolosa* Δ*fixLJ* + p*fixLJ* carrying G345S FixL and FixJ, integrated at *att*Tn7 site downstream of AK34_4894	This study
VC7102 (FixL T353M)	VC7102 with T353M mutation in native *fixL* gene using pBM_FixL_T353M	This study
VC7102 (FixL R400G)	VC7102 with R400G mutation in native *fixL* gene using pBM_FixL_R400G	This study
Δ*fixLJ* + W439S *fixLJ*/pfix- reporter	*B. dolosa* Δ*fixLJ* complemented with W439S *fixLJ* carrying p*fixK* reporter	21
Δ*fixLJ* + empty vector/p*fixK-*reporter	*B. dolosa* Δ*fixLJ* complemented with empty vector carrying p*fixK* reporter	21
Δ*fixLJ* + *fixLJ*/p*fixK-*reporter	*B. dolosa* Δ*fixLJ* complemented with *fixLJ* carrying p*fixK* reporter	This study
Δ*fixLJ* + R347H *fixLJ*/p*fixK-*reporter	*B. dolosa* Δ*fixLJ* complemented with R347H *fixLJ* carrying p*fixK* reporter	This study
Δ*fixLJ* + G345S *fixLJ*/p*fixK-*reporter	*B. dolosa* Δ*fixLJ* complemented with G345S *fixLJ* carrying p*fixK* reporter	This study

a Km, kanamycin.

**TABLE 3:** Plasmids used in this study

	Notes^*a*^	Source
pEXkm5	Km^r^, *sacB*, *gusA*	25
pTNS3	Amp^r^, helper plasmid for mini-Tn7 integration into *att*Tn7 site	53
pRK2013	Km^r^ conjugation helper	52
pSCrhaB2	Tp^r^, *ori*_pBBR1_*rhaR*, *rhaS*	69
pUC18T-mini-Tn7T-Tp	Amp^r^, Tp^r^ on mini-Tn7T; mobilizable	52
p*fixLJ*-W439S	pUC18T-mini-Tn7T-Tp carrying W439S FixL and FixJ with 670 bp upstream flanking	21
p*fixLJ*-*anc*	pUC18T-mini-Tn7T-Tp carrying FixL and FixJ with 670 bp upstream flanking	This study
p*fixLJ*-R347H	pUC18T-mini-Tn7T-Tp carrying R347H FixL and FixJ with 670 bp upstream flanking	This study
p*fixLJ*-G345S	pUC18T-mini-Tn*7*T-Tp carrying G345S FixL and FixJ with 670 bp upstream flanking	This study
pBM_FixL_T353M	pEXkm5 containing 1.5-kbp fragment encoding T353M FixL mutation	This study
pBM_FixL_R400G	pEXkm5 containing 1.5-kbp fragment encoding R400G FixL mutation	This study
p*fixK* -reporter	pSCrhaB2 carrying *B. dolosa fixK-lacZ* fusion with Km^r^ in place of Tp^r^	21
pENTR	Gateway cloning entry vector	Invitrogen
pTRX-HIS-DEST	Expression vector for response regulator Amp^r^	30
pHIS-MBP-DEST	Expression vector for histidine kinase Amp^r^	30
pENTR-FixJ	*B. dolosa* AU0158 *fixJ* cloned into pENTR	This study
pENTR-FixL	*B. dolosa* AU0158 *fixL* amino acids 329 to 851 cloned into pENTR	This study
pFixJ_Expression	*B. dolosa fixJ* cloned into pTRX-HIS-DEST for purification	This study
pFixL_W439_Expression	*B. dolosa fixL* containing W439 sequence cloned into pHIS-MBP-DEST for purification	This study
pFixL_W439S_Expression	*B. dolosa fixL* containing W439S sequence cloned into pHIS-MBP-DEST for purification	This study
pFixL_R347H_Expression	*B. dolosa fixL* containing R347H sequence cloned into pHIS-MBP-DEST for purification	This study
pFixL_G345S_Expression	*B. dolosa fixL* containing G345S sequence cloned into pHIS-MBP-DEST for purification	This study

a Amp, ampicillin; Km, kanamycin; Tp, trimethoprim.

### Genetic manipulations and strain construction.

To generate *B. dolosa* mutants carrying *fixL* mutations, we introduced the desired mutations using the Q5 site-directed mutagenesis kit (New England Biolabs) into p*fixLJ* (21). p*fixLJ* contains the AU0158 *fixLJ* sequence along with 670 bp upstream within the pUC18-mini-Tn7-Tp back bone, allowing for stable chromosomal integration at an *att*Tn7 site (24, 52), and was renamed p*fixLJ*-W439S for this study. Point mutations were verified by Sanger sequencing. The *B. dolosa fixLJ* complementation vectors and the corresponding empty vector controls were conjugated into the AU0158 *fixLJ* deletion mutant with pRK2013 and pTNS3 using published procedures (21). Conjugants were selected for by plating on LB agar containing trimethoprim (1 mg/ml) and gentamicin (50 μg/ml). Insertions into the *att*Tn7 site downstream of AK34_4894 were confirmed by PCR. To generate *B. multivorans* VC7102 mutants carrying *fixL* mutations, we introduced the desired *fixL* mutations in the native *fixL* gene using the suicide plasmid pEXKm5 (25). Briefly, approximately 1 kbp upstream and downstream of the desired mutation were PCR amplified, as was pEXKm5, and then the two fragments were joined using the NEBuilder HiFi DNA assembly master mix (New England BioLabs) per the manufacturer’s protocol. The plasmids were Sanger sequenced and transformed into RHO3 E. coli and then conjugated into *B. multivorans* VC7102 (24, 53). Conjugants were selected for on LB with kanamycin at 1 mg/ml. To resolve merodiploidy, conjugants were counterselected against by plating on LB with 15% (wt/vol) sucrose and incubated for 2 days at 30°C. Clones were screened for the introduction of the desired mutation by PCR and Sanger sequencing.

Expression vectors for FixL and FixJ His_6_-tagged proteins were generated using the Gateway high-throughput recombinational cloning system (Invitrogen). The entire *fixJ* gene or *fixL* amino acids 329 to 851 (lacking transmembrane domains) were amplified from *B. dolosa* AU0158 and cloned in pENTR. Gateway LR clonase reactions were used to move *fixL* or *fixJ* into pHIS-MBP-DEST or pTRX-HIS-DEST, respectively, for expression (30). *fixL* mutations were introduced using a Q5 site-directed mutagenesis kit (New England Biolabs), and sequences were confirmed by Sanger sequencing. To generate *fixK* reporter strains, a *fixK-lacZ* fusion reporter was conjugated into *B. dolosa* constructs as previously described (21).

### Bacterial invasion assays.

The ability of *B. dolosa* to invade and persist within macrophages was determined using published protocols (21). Human THP-1 monocytes were differentiated into macrophages by seeding 1 ml into 24-well plates at 8 × 10^5^ cells/ml with 200 nM phorbol 12-myristate 13-acetate (PMA). THP-1-derived macrophages were infected with log-phase grown BCC washed in RPMI three times at ∼2 × 10^6^ CFU/well (multiplicity of infection [MOI] of ∼10:1). Plates were spun at 500 × *g* for 5 min to synchronize infection and were then incubated for 15 min to 2 h at 37°C with 5% CO_2_. To determine the total number of bacteria, wells were treated with 100 μl of 10% Triton X-100 lysis buffer (final concentration, 1% Triton X-100), serially diluted, and plated to enumerate the number of bacteria. To determine the number of intracellular bacteria, separate infected wells were washed two times with phosphate-buffered saline (PBS) and then incubated with RPMI + 10% heat-inactivated FCS with kanamycin (1 mg/ml) for 1 to 24 h. Monolayers were washed three times with PBS and lysed with 1% Triton X-100, serially diluted, and plated to enumerate the number of bacteria. In some experiments, THP-1 cells were differentiated into macrophage-like cells using PMA described above on 8-well chamber slides (Lab-Tek) and were infected with 2 × 10^5^ CFU for 2 h, and cells were washed with PBS 4 times and stained and fixed with a Hema 3 stain set (Fisher Scientific) per the manufacturer’s protocol.

### Murine model of pneumonia.

All animal protocols and procedures were approved by the Boston Children’s Hospital Institutional Animal Care and Use Committee (assurance number A3303-01). The specific protocol number is 18-01-3617R. All animal protocols are compliant with the NIH Office of Laboratory Animal Welfare, the Guide for the Care and Use of Laboratory Animals, the US Animal Welfare Act, and the PHS Policy on Humane Care and Use of Laboratory Animals. Female C57BL/6 mice 6 to 8 weeks of age were obtained from Taconic Biosciences. Mice were maintained at the animal facilities at Boston Children’s Hospital. Mice were anesthetized with ketamine (100 mg/kg) and xylazine (13.3 mg/kg) given intraperitoneally. While the mice were held in dorsal recumbency, 10 μl of inoculum was instilled in each nostril (20 μl total). The inoculum consisted of log-phase *B. dolosa* washed in PBS and diluted to a concentration of ∼2 × 10^10^ CFU/ml (4 × 10^8^ CFU/mouse). Mice were euthanized 7 days after infection by CO_2_ overdose, and lungs and spleens were aseptically removed. Lungs and spleens were weighed and placed into 1 ml of 1% proteose peptone in water, homogenized, and then serially diluted and plated on oxidation/fermentation-polymyxin-bacitracin-lactose (OFPBL) plates.

### Soil survival assays.

Top soil (coast of Maine) was autoclaved twice after removing stones and other large debris. One gram of soil was then placed into a 14-ml polystyrene tube. *B. dolosa* was grown overnight in M9 medium supplemented with 20 μM succinate, 2 μM MgSO_4_, and 0.1 μM CaCl_2_ at 37°C with shaking at 200 rpm. *B. dolosa* inocula were then washed in M9 and diluted to 1 to 3 × 10^8^ CFU/ml in M9. Two-hundred microliters of each bacterial suspension was added to the tubes containing 1 g of soil. Soil tubes were incubated for 10 days at 30°C without agitation. After that, 1 ml of 1% protease peptone in water with 1% Triton X-100 was added to each tube, and the tubes were vigorously vortexed. Soil was allowed to settle for 15 min, after which bacteria were enumerated by serial plating on Trypticase soy agar (TSA) plates.

### Biofilm formation.

The ability to form biofilms on polyvinyl chloride (PVC) plates was determined using published methods (54). Briefly, overnight cultures were diluted in Trypticase soy broth (TSB) with 1% glucose and pipetted into wells of a 96-well PVC plate. Plates were incubated for 48 h at 37°C, and unattached bacteria were washed with water. Biofilms were stained with 0.5% crystal violet, excess stain was washed away, and stain was solubilized with 33% acetic acid. The solution was transferred to a flat-bottom plate, and then biofilm amount was quantified by measuring optical density at 540 nm (OD_540_).

### Motility assay.

The ability of *B. dolosa* to swim was measured in low-density LB agar using a modification of published methods (55). Briefly, 10 μl of overnight *B. dolosa* culture was plated in the center of low-density (0.3% agar) LB plates. Plates were incubated agar side down for 48 h at 37°C when swimming diameter was measured. The swimming diameter was visible throughout the thickness of the agar.

### RNA-seq.

RNA was isolated from log-phase *B. dolosa* (two biological replicates per construct) using the Ribopure bacterial RNA purification kit (Ambion) per the manufacturer’s protocol, and contaminant DNA was removed using DNase. RNA was processed, and libraries were generated as previously published (21). Samples were sequenced using single-end 50 bp reads using the Illumina HiSeq platform. Data analysis was done using Galaxy (https://usegalaxy.org) (56). Reads (9 to 12 million reads per replicate) were trimmed using the Trimmomatic tool (57) and mapped to the *B. dolosa* AU0158 genome (GenBank assembly accession number GCA_000959505.1) (58) using BowTie2 with very sensitive local preset settings (59). Differentially expressed genes were identified using CuffDiff using the Benjamini-Hochberg procedure to determine the *q* value (*P* value corrected for multiple comparisons) (60). Reads were deposited to BioProject PRJNA579568. GO terms that were enriched among genes that were differentially regulated were identified using GoSeq using a Wallenius approximation and a Benjamini-Hochberg test to determine a corrected *P* value for multiple comparisons (61). GO terms that were considered to be enriched had an adjusted *P* value of <0.05.

### qRT-PCR.

cDNA was synthesized from 2 μg of RNA using the ProtoScript II first strand cDNA synthesis kit (New England BioLabs) per the manufacturer’s protocol. cDNA was cleaned using a QIAquick PCR purification kit (Qiagen). Oligonucleotides to amplify *gyrB*, *rpoD*, and *fliC* were previously published (21), and *motA* was amplified using 5′-GTGAAGATCGGGCTCTTGT-3′ and 5′-GGACGTCTATATGGAGCTGATG-3′. Genes were amplified using oligos FastStart essential DNA green master mix (Roche) per the manufacturer’s protocol. Expression was determined relative to *B. dolosa* AU0158 carrying the evolved *fixL* allele (encoding FixL W439S) normalized by *gyrB* (AK34_3072) or *rpoD* (AK34_4533) expression using the threshold cycle (ΔΔ*C_T_*) method (62). Both *gyrB* and *rpoD* had similar expression by RNA-seq between AU0158 and the *fixLJ* deletion mutant, and these genes have been used to normalize expression in B. cenocepacia in other studies (63, 64).

### Protein expression and purification.

FixL and FixJ expression vectors were transformed into E. coli BL21(DE3) cells, and protein was expressed and purified using nickel affinity columns following published protocols (30).

### In vitro phosphorylation.

Autophosphorylation and phosphotransfer assays were done as previously described (65). Briefly, FixL variants were used at a final concentration of 2.5 μM mixed with a final concentration of 5 mM MgCl_2_, 0.5 mM ATP, and 2.5 μCi of [γ^32^P]ATP (stock of 6,000 Ci/mmol, 10 mCi/ml; PerkinElmer). Autophosphorylation reactions were performed at 30°C with ambient oxygen and were stopped at the indicated time points by the addition of 4× sample buffer (200 mM Tris-HCl at pH 6.8, 400 mM dithiothreitol (DTT), 8% SDS, 0.4% bromophenol blue, 40% glycerol). For phosphotransfer assays, FixL variants were autophosphorylated using the above parameters at 30°C for 15 min and then incubated with reaction mixtures containing the response regulator FixJ and MgCl_2_ at final concentrations of 5 μM and 5 mM, respectively. Phosphotransfer reactions were run at 30°C with ambient oxygen. Reactions were stopped at the indicated time points with the addition of 4× sample buffer. Samples were then run on an “any kD” Bio-Rad mini-protean TGX gels for 50 min at 150 V. Gels were exposed to phosphor screens for 4 to 5 h so that phosphorylated protein bands could be observed. Screens were imaged using the Typhoon-FLA9500 imager with a “phosphor” setting and a resolution of 50 μm. Band intensity of phosphorylated proteins was quantified using ImageJ.

### Reporter assay.

BCC carrying a *fixK-lacZ* reporter plasmid was grown overnight in LB with kanamycin (1 mg/ml). Cultures were subcultured in LB in ambient oxygen or LB that had been degassed in the CampyGen gas generating system (Thermo Fisher). Cultures were grown in ambient oxygen with shaking (200 rpm) at 37°C or within the CampyGen gas generating system at 37°C for 4 to 6 h. The level of *fix* pathway-driven LacZ activity was measured by determining Miller units following published procedures (66).

### c-di-GMP quantification.

*B. dolosa* constructs were grown to stationary phase when 50-ml aliquots were spun down, and c-di-GMP was extracted using ice-cold extraction buffer (methanol:acetonitrile:distilled water, 40:40:20 + 0.1 N formic acid). c-di-GMP levels were measured using mass spectroscopy as previously described (67).

### Data availability.

RNA-seq reads have been deposited to BioProject under accession number PRJNA579568 (https://www.ncbi.nlm.nih.gov/bioproject/579568).
