# Supplementary figures and images for: Evolution towards Virulence in a Burkholderia Two-Component System
Source: mBio. 2021 Aug 10;12(4):e01823-21. doi: 10.1128/mBio.01823-21 (PMC8406202; doi:10.1128/mBio.01823-21)

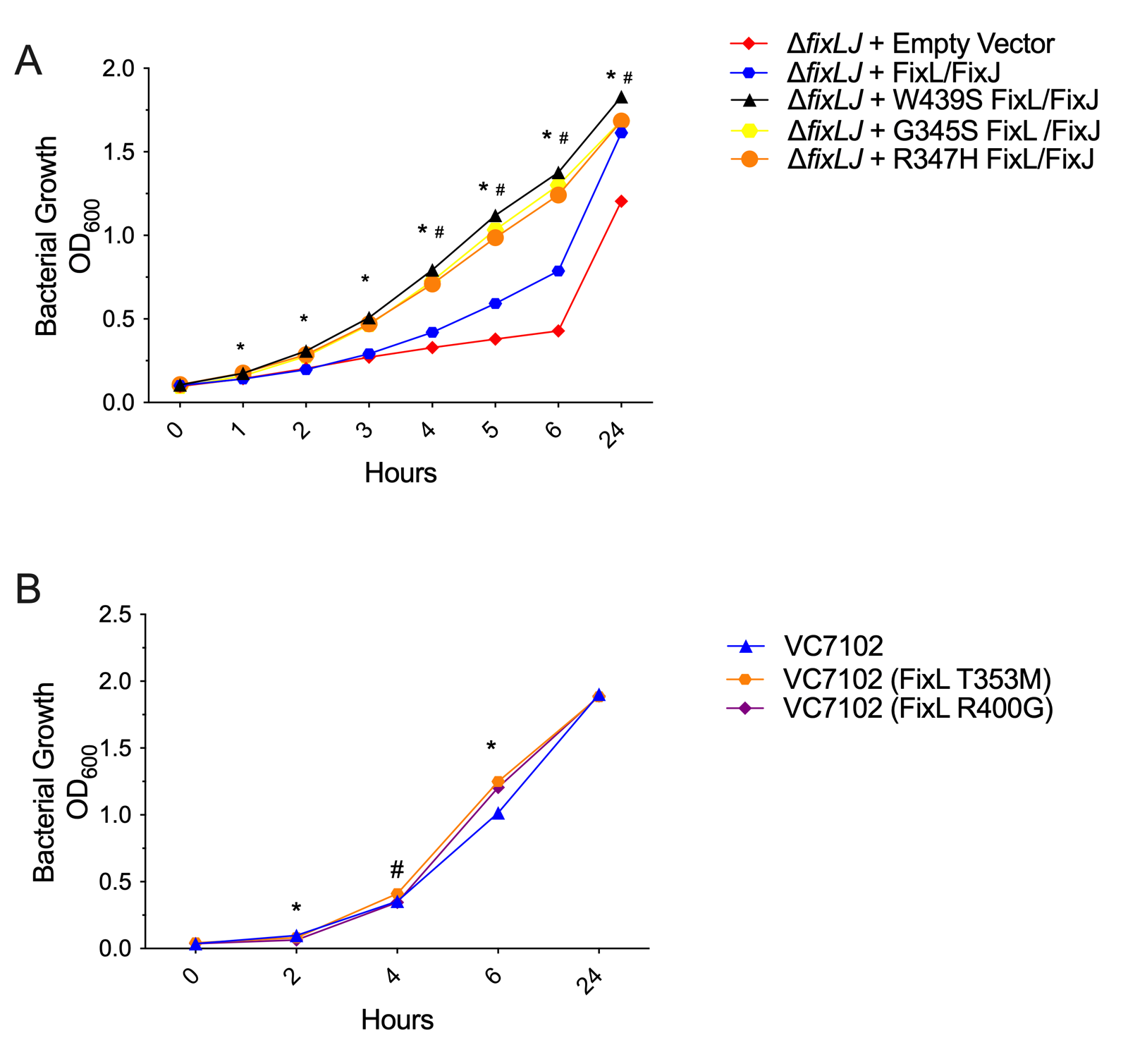

Supplement: FIG S1 [file mbio.01823-21-sf001.tif]

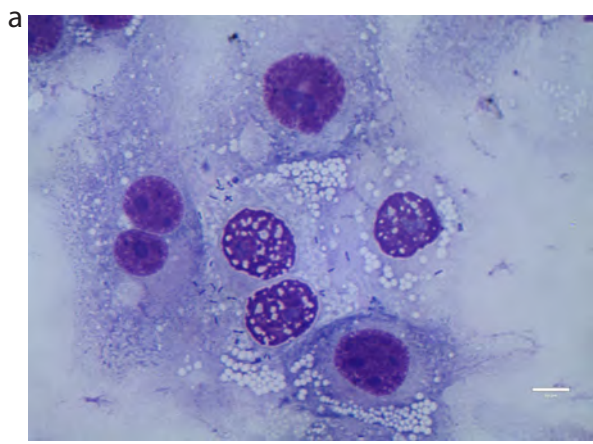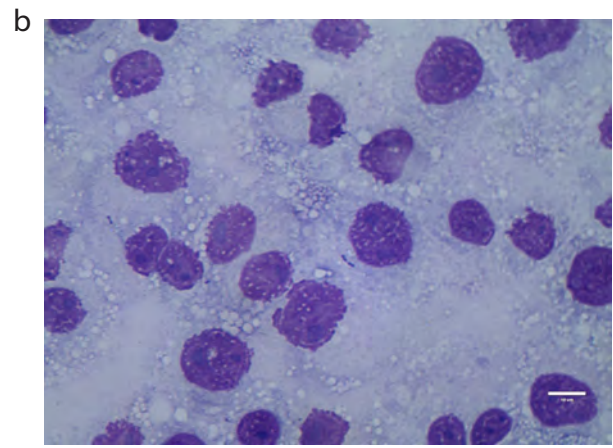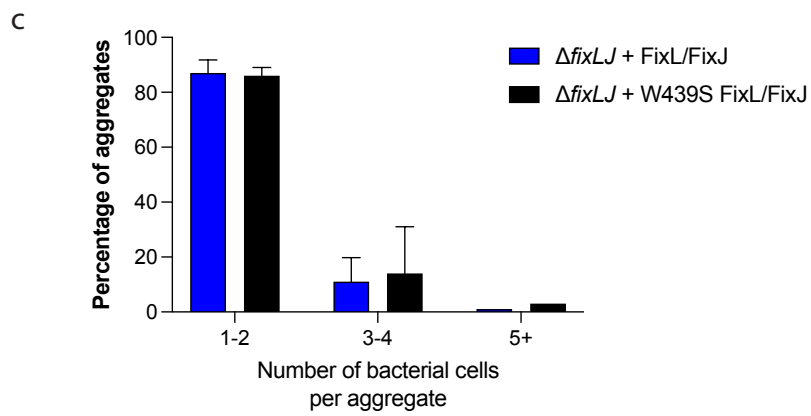

Supplement: FIG S2 [file mbio.01823-21-sf002.pdf]

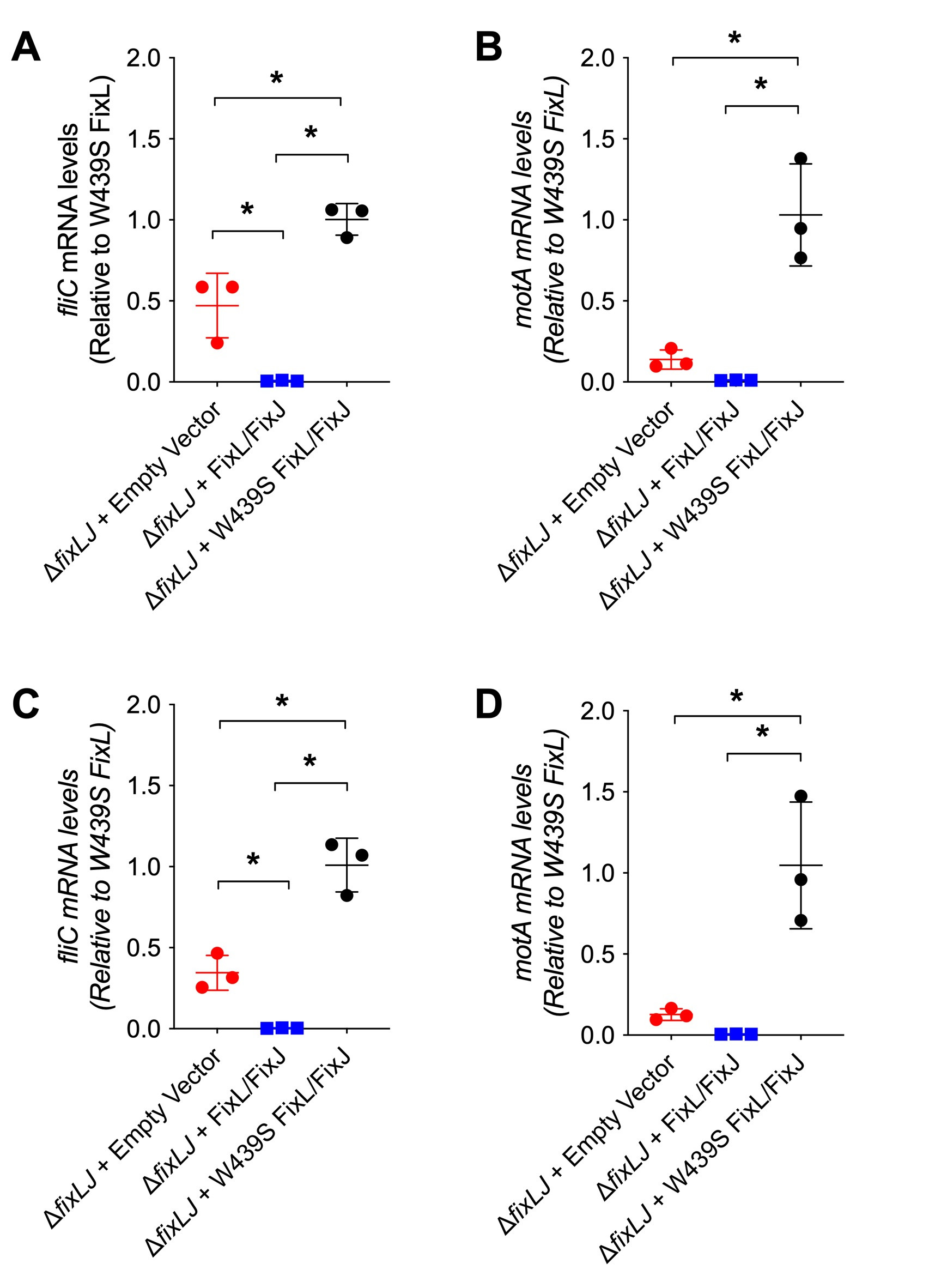

Supplement: FIG S3 [file mbio.01823-21-sf003.tif]

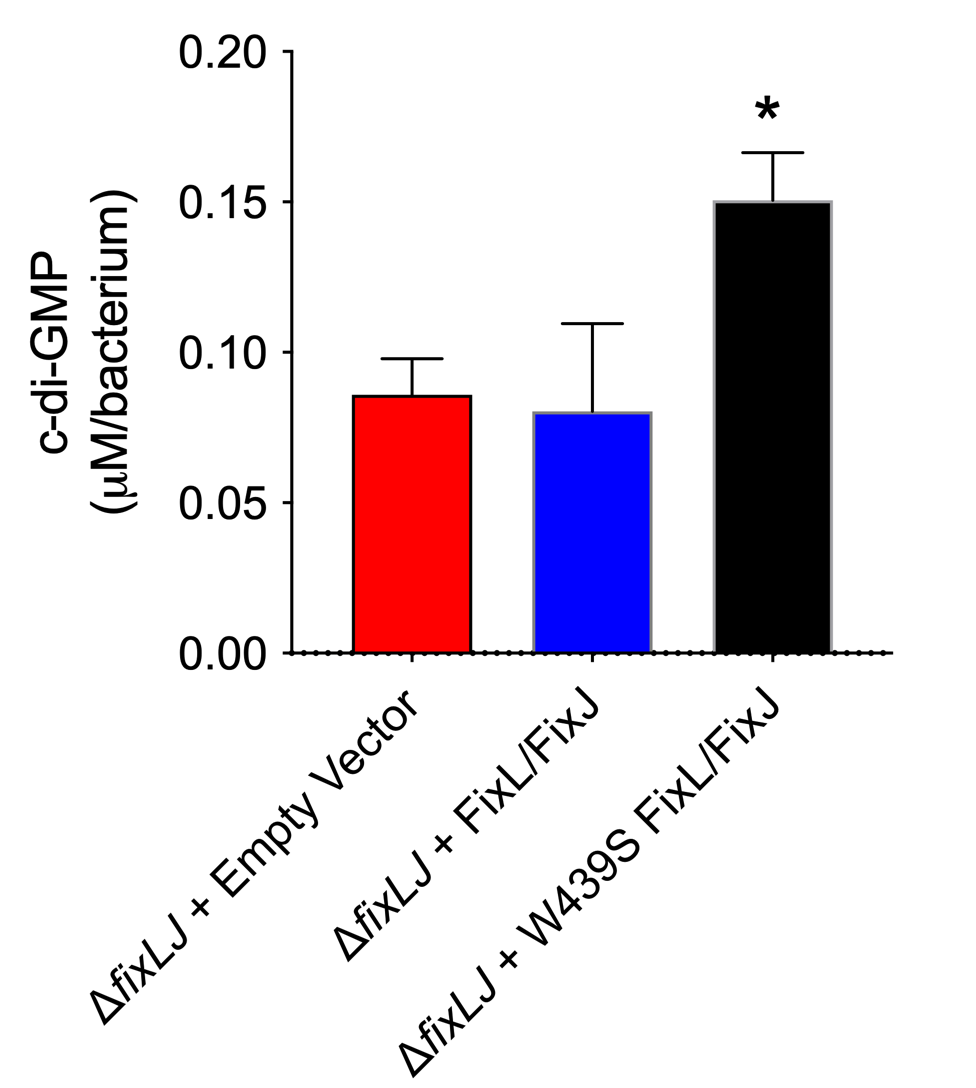

Supplement: FIG S4 [file mbio.01823-21-sf004.tif]

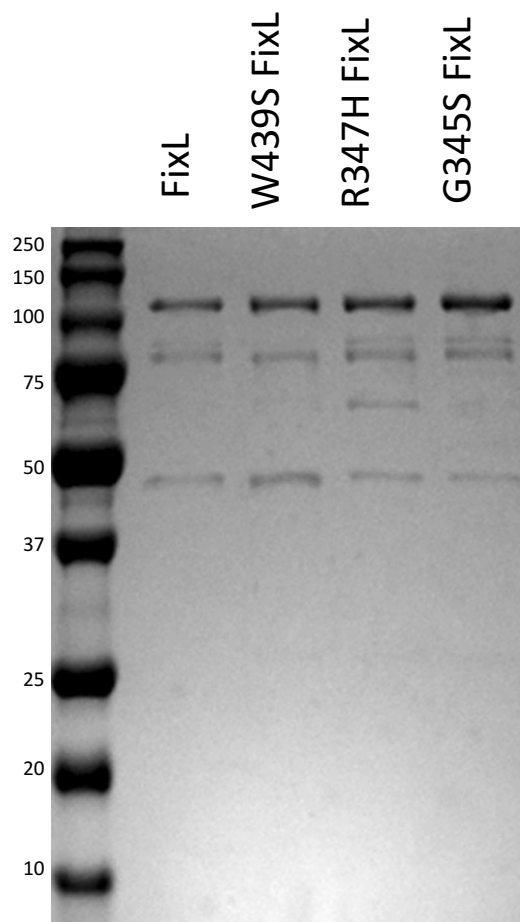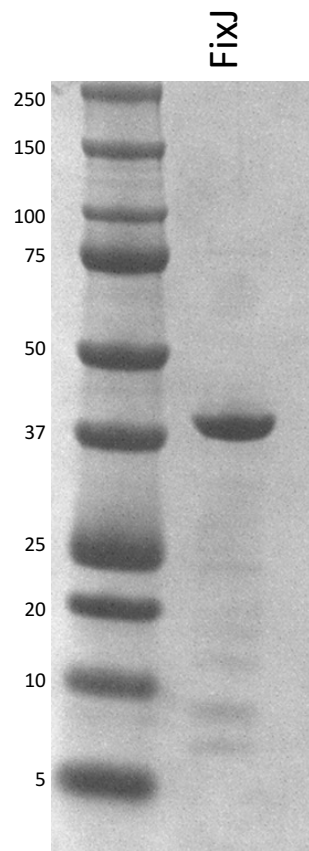

Supplement: FIG S5 [file mbio.01823-21-sf005.pdf]

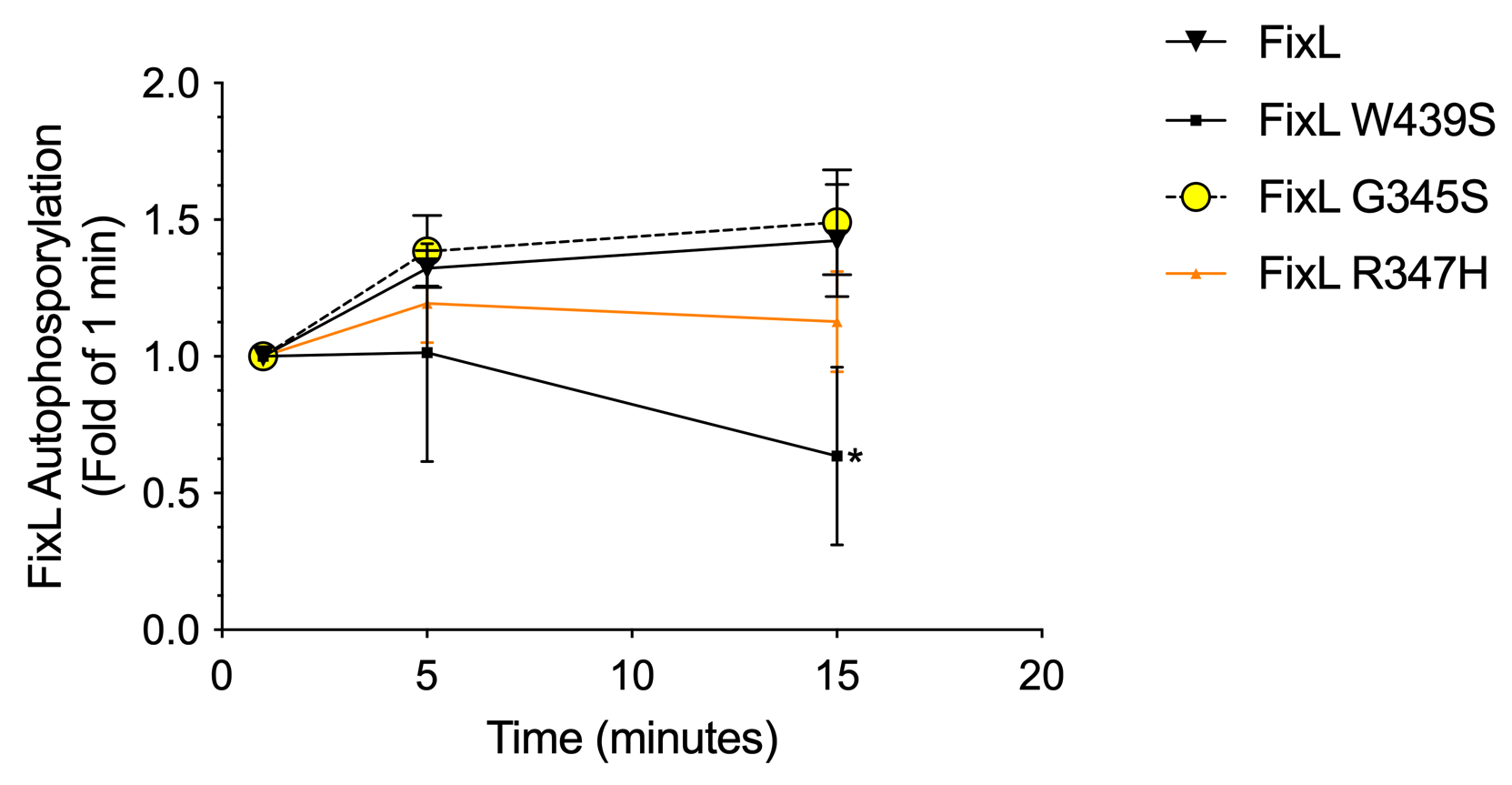

Supplement: FIG S6 [file mbio.01823-21-sf006.tif]
