# Supplementary material for: Evolution towards Virulence in a Burkholderia Two-Component System
Source: mBio. 2021 Aug 10;12(4):e01823-21. doi: 10.1128/mBio.01823-21 (PMC8406202; doi:10.1128/mBio.01823-21)
Supplement: TABLE S2 [file mbio.01823-21-st002.docx]

**Table S2.** Selected genes of interest that are significantly differentially expressed in *B. dolosa* carrying ancestral *fixL* sequence relative *B. dolosa* carrying evolved *fixL* sequence variant.

| **Category/ Gene name (genome designation)** | **Description** | **Fold change in *B. dolosa* carrying evolved FixL sequence** |
| --- | --- | --- |
| c-di-GMP metabolism |  |  |
| AK34_137 | GGDEF and EAL domain containing protein | 3.5 |
| AK34_1958 | *cpdA* homolog  GGDEF and EAL domain containing protein | 8.5 |
| AK34_5467 | Response regulator containing an HD-GYP domain | 9.8 |
| *wsp* system |  |  |
| AK34_4625 | *wspA* | -2.9 |
| AK34_4626 | *wspHRR* | -4.0 |
| AK34_4623 | *wspC* | -3.0 |
| AK34_4621 | *wspE* | -3.0 |
| Others |  |  |
| AK34_4608 | AraC-like Transcriptional regulator | -94.6 |
| AK34_3040 | *cidA*  Holin-like protein | -63.9 |
| AK34_3041 | *cidB*  CidA-associated membrane protein | -41.6 |
